# Fabrication of Single Crystal Gallium Phosphide Thin Films on Glass

**DOI:** 10.1038/s41598-017-05012-w

**Published:** 2017-07-05

**Authors:** Hal Emmer, Christopher T. Chen, Rebecca Saive, Dennis Friedrich, Yu Horie, Amir Arbabi, Andrei Faraon, Harry A. Atwater

**Affiliations:** 1 Applied Physics and Materials Science, California Institute of Technology, Pasadena, 91125 USA; 2 Joint Center for Artificial Photosynthesis, California Institute of Technology, Pasadena, CA 91125 USA; 3 Institute for Solar Fuels, Helmholtz-Zentrum Berlin, für Materialien und Energie GmbH, Hahn-Meitner-Platz 1, 14109 Berlin, Germany

## Abstract

Due to its high refractive index and low absorption coefficient, gallium phosphide is an ideal material for photonic structures targeted at the visible wavelengths. However, these properties are only realized with high quality epitaxial growth, which limits substrate choice and thus possible photonic applications. In this work, we report the fabrication of single crystal gallium phosphide thin films on transparent glass substrates via transfer bonding. GaP thin films on Si (001) and (112) grown by MOCVD are bonded to glass, and then the growth substrate is removed with a XeF_2_ vapor etch. The resulting GaP films have surface roughnesses below 1 nm RMS and exhibit room temperature band edge photoluminescence. Magnesium doping yielded p-type films with a carrier density of 1.6 × 10^17^ cm^−3^ that exhibited mobilities as high as 16 cm^2^V^−1^s^−1^. Due to their unique optical properties, these films hold much promise for use in advanced optical devices.

## Introduction

The realization of high quality gallium phosphide films on arbitrary substrates via transfer fabrication is an important step towards creating novel advanced optical devices. Gallium phosphide is an important photonic material due to its high refractive index (n > 3 throughout the visible range^1^) and high transparency across much of the visible range. Absorption is very low below the indirect bandgap at 2.26 eV and still weak (α < 3500 cm^−1^) below the L-transition at 2.5–2.6 eV^1–3^. In fact, GaP has the highest bandgap of all commonly explored high refractive index semiconductor materials with n > 3 throughout the visible range^4^. These properties make GaP well suited for many advanced visible and infrared photonics applications requiring high refractive index contrast, such as high contrast gratings^5^ and nanophotonic metasurfaces^6^.

High index contrast enables strong light confinement due to the change in amplitude of the electric field at an interface between materials of different index^7^. This confinement allows the use of subwavelength structures to guide and otherwise interact with light, necessary for the fabrication of highly integrated photonic devices^8^. The confinement within a slot waveguide, for example, is given by ${n}_{H}^{2}/{n}_{S}^{2}$, where ${n}_{H}\,\mathrm{and}\,{n}_{S}$ are the refractive indices of the high index material and the substrate, respectively. The structures explored by Almeida at el. used the Si-air and Si-SiO_2_ materials systems, useful with low losses only beyond the Si band gap; in this case, the telecom wavelength 1.55 μm was discussed. Use of gallium phosphide in similar structures would extend the useful wavelength for this type of device well into the visible range.

Beyond waveguides, a diverse array of photonic devices exploit high index contrast. Metasurfaces created with high index contrast materials can enable control over wave amplitude, phase and polarization^9^ in subwavelength antennas, yielding flat filters^10^ and lenses^11^ created via systematic variation of the dimensions of subwavelength resonators. GaP also has a high second-order non-linear optical coefficient^12^, useful in photonic devices for parametric down-conversion, second harmonic generation^13^ and sum/difference frequency generation^14^. Gallium phosphide resonant cavities used to measure non-linear properties have previously been fabricated on free standing GaP membranes, grown on a sacrificial AlGaP layer^15^. The transfer of thin GaP films from GaP/Si allows the fabrication of GaP films on large-area, low-cost substrates with a simpler and less expensive fabrication scheme. The growth of GaP on Si is well understood; and while limitations exist in the achievable surface roughness^16^ and density of lattice defects in the form of antiphase domains, sufficient material quality for optical devices has been achieved^13^. The substrate removal process draws from the well-established epitaxial lift-off technique^17, 18^; here, substrate reuse is not critical, because the substrate is silicon as opposed to expensive GaAs.

**Figure 1:**
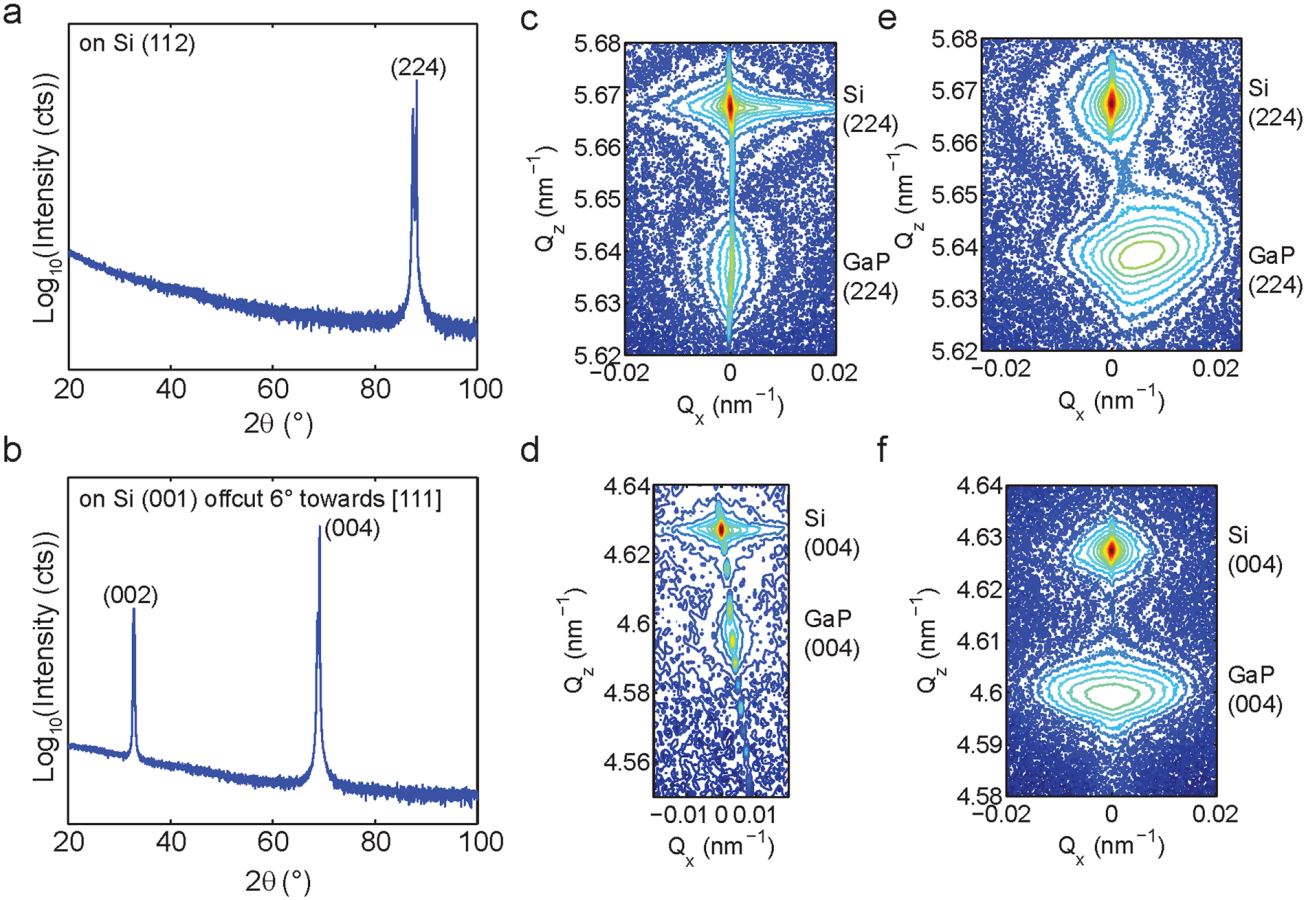
High resolution x-ray diffractometry of GaP films grown on Si. (**a**), (**b**) x-ray diffraction spectra of ~200 nm thick GaP films grown on (112) and (001) offcut 6° towards [111] substrates, respectively. The amorphous background is due to the use of a glass slide onto which the 1 × 1 cm sample is mounted. (**c**), (**d**) reciprocal space maps of ~50 nm thick films. (**e**), (**f**) reciprocal space maps of ~200 nm thick films.

Epitaxial growth of GaP was obtained on both (112) and (001)−6° offcut silicon substrates, as verified by X-ray diffraction. High resolution x-ray diffraction spectra with reciprocal space maps are shown in Fig. 1. Reciprocal space maps of films ~ 50 nm thick, below the critical thickness for GaP grown on silicon, show Laue oscillations and film peak widths (36.2 arcseconds) matching the width of the substrate peak (36 arcseconds). This indicates that thin films are not relaxed and have low crystalline defect density. Reciprocal space maps of films above the critical thickness show that films were fully relaxed when grown on (112) substrates and 96% relaxed when grown on the offcut (001) substrates. At thicknesses higher than the critical thickness, such as those demonstrated in this paper, threading dislocations are expected and observed for both substrate orientations^19^.

**Figure 2:**
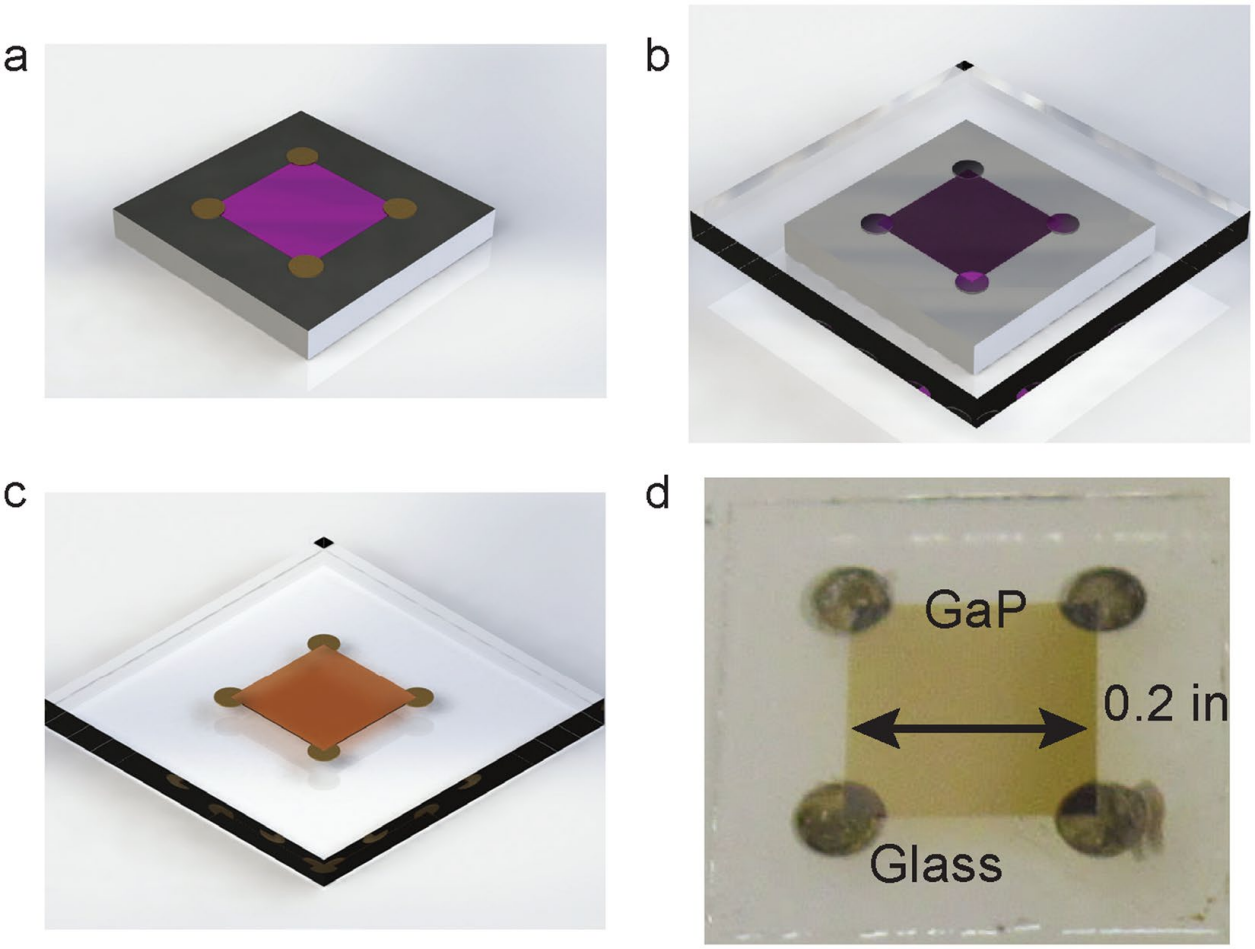
Schematic of the substrate removal process. (**a**) The GaP film is first etched to form a square 0.2 inches on a side. Metal contacts are then deposited and annealed on the corners, extending off the edge. (**b**) The sample is then bonded to a glass slide and (**c**) the silicon substrate etched away with XeF_2_, leaving the GaP film and metal contacts. (**d**) photograph of a typical contacted, substrate removed GaP film.

A schematic of the substrate removal process is shown in Fig. 2. First, a mesa etch was performed on the GaP films, defining a 0.2 inch square appropriate for Hall effect measurements. The mesa etch also created a sharp edge which allowed thickness measurements to be performed using a stylus contact profilometer. Measured growth rates varied very little; all thickness measurements were within 10% of the target thickness. Following the mesa etch, contacts were formed at sample corners. The metallization step not only contacts the film for electronic measurements, but also acts as a spacer to define the thickness of the adhesive layer that holds the GaP film to the glass handle. Samples were then attached to glass handle substrates with an adhesive interlayer. Two different interlayers were explored, which had different properties. Crystalbond 509 wax was fast and easy to apply – the sample was simply pressed onto the substrate on a hotplate. However, this interlayer resulted in buckled films that, while acceptable for electronic measurements, had relatively poor optical quality. Optically smooth films were achieved by using an SU-8 2002 interlayer between GaP and glass and transfer with a wafer bonding tool^20^. Silicon wafers were then removed with a pulsed XeF_2_ vapor etcher. This dry silicon etch was chosen for the substrate removal process due to its high selectivity for silicon over glass, most organics, and most metals^21^ and generally gentle etching action, which ensures good adhesion between the film and the glass handle substrate.

**Figure 3:**
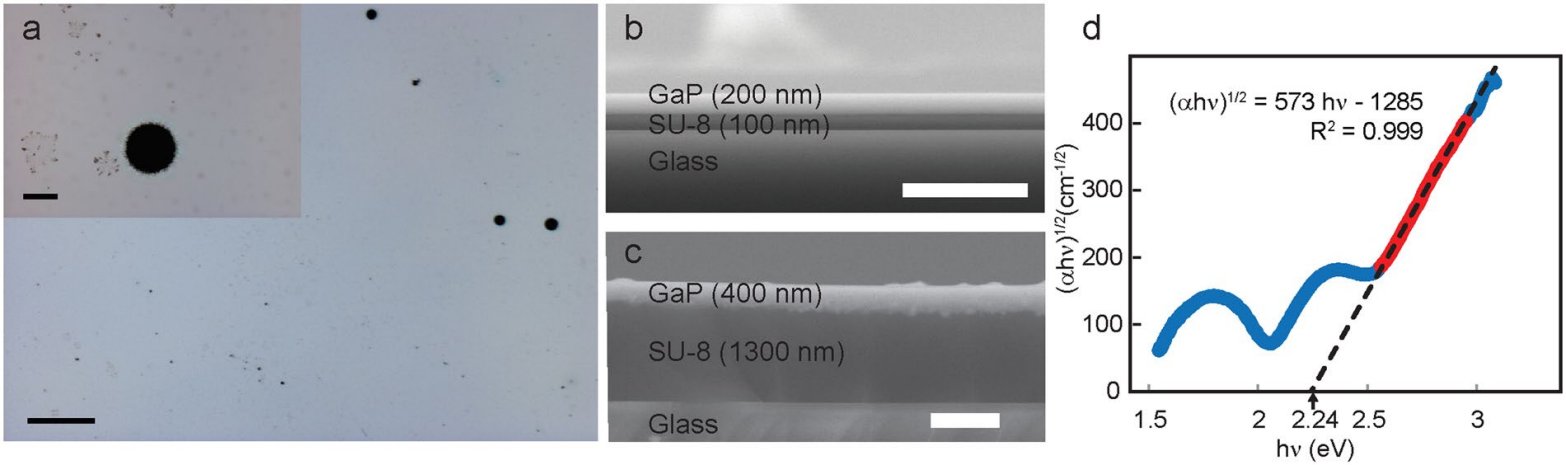
Micrographs and transmission spectrum of substrate removed GaP film. (**a**) Optical micrograph showing some typical defects in the films. The scale bar is 200 μm in the large image and 20 μm in the inset. (**b**) Cross sectional scanning electron microscope image of a thin (200 nm) GaP film bonded to glass with a thin interlayer. This particular film did not have metal contacts, and as a result, the interlayer was only 100 nm thick. The scale bar is 1 μm. (**c**) Cross sectional scanning electron microscope image of a thick (400 nm) GaP film bonded to glass with a thick interlayer (1300 nm), determined by the thickness of the metal contacts. The scale bar is 1 μm. (**d**) Tauc plot generated from normal incidence transmission measurements of a substrate removed GaP film. A linear fit was generated from the red data points with bounds selected to maintain R = 0.999, and the fit indicates a bandgap of 2.24 eV.

An optical microscope image showing a pinhole defect in the film is shown in Fig. 3a, and two SEM cross sections of GaP films of different thicknesses bonded with thin and thick interlayers are shown in Fig. 3b and c. The pinhole defects were present in the original GaP film as grown on silicon, likely resulting from particulate contamination occurring prior to or during growth. The thickness of the interlayer can be increased by adding metal contacts to the film corners; films with our typical Hall measurement contacts had an interlayer thickness of approximately 1 μm.

Optical transmission measurements were performed on the GaP/wax/glass structure in a spectrophotometer equipped with an integrating sphere as described elsewhere^22^. Transmission measurements were normalized to a reference spectrum consisting of wax and glass. A Tauc plot (αhν^1/2^ vs photon energy, where α is the absorption coefficient, hν is photon energy, and ½ is used for an indirect gap^23^) was generated from the normal incidence transmission data and used to extract the bandgap, as shown in Fig. 3d. The Tauc plot is linear near the expected band edge, with a good linear fit, indicating an indirect bandgap^23^. The extents of the fit were selected to maintain an R^2^ value of 0.999. We can extrapolate this curve to extract a bandgap of 2.24 eV, in good agreement with the accepted value of 2.26 eV^2^. The features in the transmission data at photon energies below the bandgap result from Fresnel reflections between the GaP film, wax, and glass.

**Figure 4:**
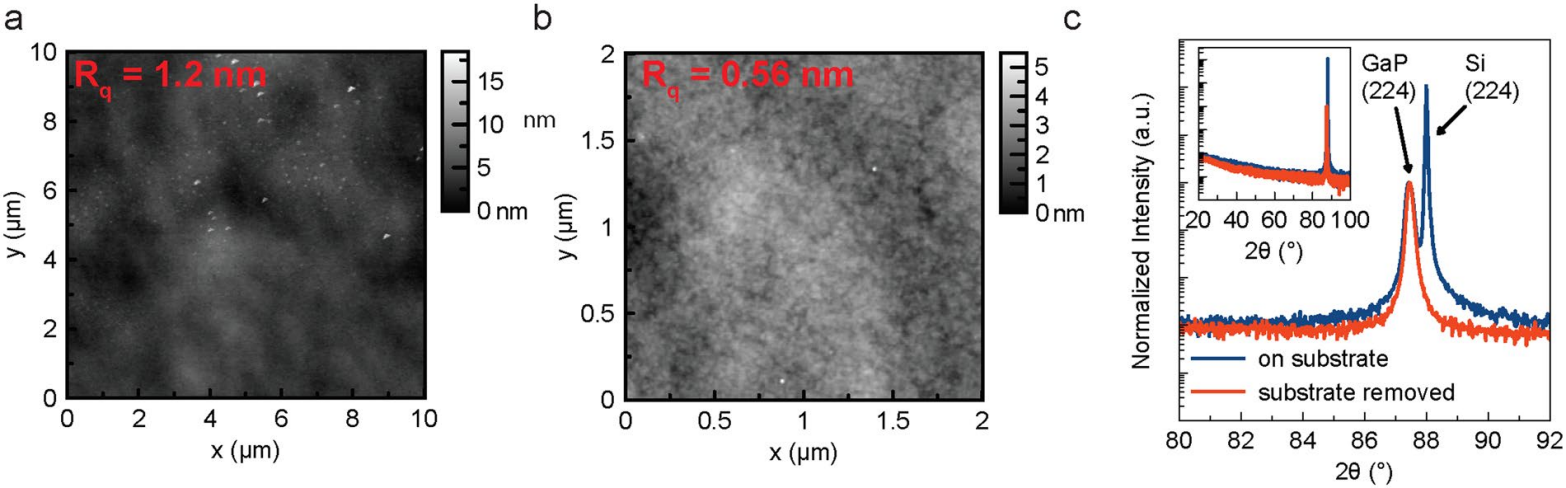
Atomic force micrographs and x-ray diffraction spectra of GaP films. Atomic force microscopy images of the GaP film top surface following growth on silicon (**a**), and following substrate removal (**b**). Note that the top surface following substrate removal is the surface which had previously been in contact with silicon in the as-grown film on substrate. (**c**) x-ray diffraction spectra of the films following substrate removal show that excellent crystallinity is maintained.

Atomic force microscopy measurements of both surfaces, before and after the substrate removal process, are shown in Fig. 4(a) and (b). The top surface RMS roughness varied slightly based on the thickness and growth conditions of the film, but roughness as low as 1 nm were measured. The bottom surface, which became the top surface following substrate removal, was reliably smooth with RMS roughness below 1 nm.

The crystallinity of GaP films following substrate removal was confirmed using X-ray diffraction. Samples were measured before and after the substrate removal process, and as expected, the crystalline, oriented films maintain their crystallinity following substrate removal, as shown in Fig. 4c. Similarly, samples from poor growths which resulted in non-oriented GaP films maintained their orientation following substrate removal.

Hall measurements were performed on transferred films in order to measure the effectiveness of doping using cyclopentadienyl-magnesium during MOCVD growth. It was necessary to perform these measurements on transferred samples to avoid the influence of the silicon substrate on the measurement^24^. Hole concentrations in the mid 10^18^ cm^−3^ range were achieved with a mobility of approximately 10 cm^2^V^−1^s^−1^, resulting in a champion resistivity of 0.12 Ω-cm. A champion mobility of 16 cm^2^V^−1^s^−1^ was measured in a sample with a carrier density of 1.6 × 10^17^ cm^−3^. The carrier densities and mobilities achieved are both slightly below, but within an order of magnitude of, the best results reported for Mg doping of MOCVD-grown GaP films, achieved on AlGaInP substrates^25^.

Photoluminescence measurements were performed on transferred GaP films. We found that substrate removal was necessary to extract enough signal to perform measurements. This was expected, since gallium phosphide has a high index of refraction (n ~ 4 near its emission peaks), well matched to the silicon substrate, which absorbs strongly at these wavelengths. Therefore, emitted photons which reach the back surface will be transmitted to the silicon substrate and absorbed with a high probability. We note that considering the solid angle of the front escape cone, one expects only 1.6% of the total luminescence emission to escape from a GaP/Si structure.

**Figure 5:**
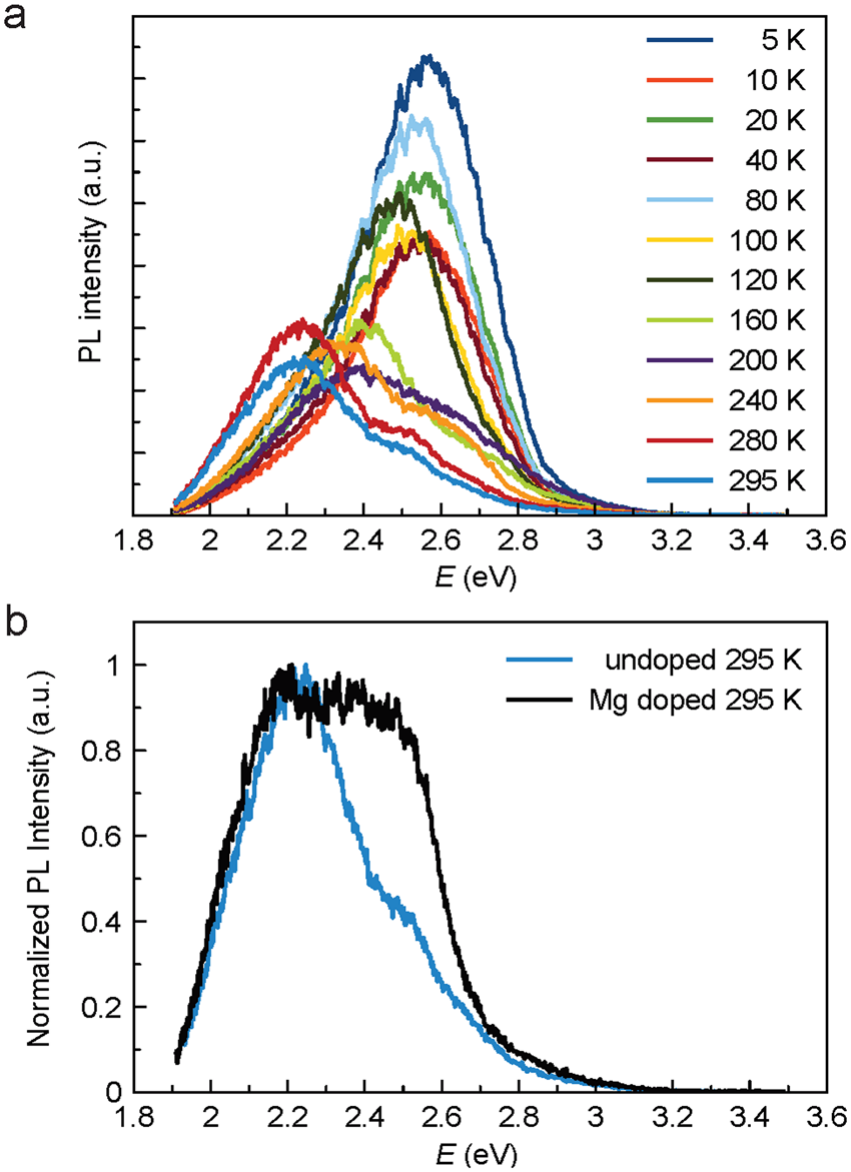
Photoluminescence spectra of GaP films. (**a**) temperature dependent photoluminescence spectra of an undoped, substrate removed GaP film, showing the primary peak at the indirect bandgap at room temperature and a shift towards the secondary gap at lower temperatures. (**b**) in the Mg doped sample, the peak at the indirect bandgap broadens and shifts, indicating states near the band edge.

Photoluminescence spectra for an undoped sample are shown in Fig. 5a. At low temperatures, the primary emission peak for both doped and undoped samples was located at approximately 2.59 eV. As the temperature increases towards room temperature, the undoped sample develops a second peak at approximately 2.26 eV, which corresponds to the primary indirect energy gap^2^. As shown in Fig. 4(b), in a heavily doped sample, good fits can be generated by considering the indirect gap peak either broadening from a full width half max of 0.34 eV to 0.38 eV, or shifting to 2.15 eV. Regardless of the fitting procedure used, this change was found to correspond to emission from dopant states close to the band edge.

In conclusion, single-crystal GaP (001) films bound to glass with a transparent interlayer were fabricated by a transfer/wafer bond/Si substrate removal process. These films had excellent optical, mechanical, and electronic properties. Due to the distinctive properties of GaP, these films open the door for fabrication of a wide variety of thin film photonic devices.

## Methods

### Gallium Phosphide Growth

Gallium phosphide was grown on silicon (001)-6º and (112) substrates using a 1 × 3-inch Thomas Swan Epitor II MOCVD system with a close-coupled showerhead. A two step growth process was used, consisting of a low temperature (450 °C) atomic layer deposition-like nucleation layer and high temperature (575 °C) growth^26^. The metalorganic precursors used were triethylgallium (63 μmol min^−1^) and tertiarybutylphosphine (3205 μmol min^−1^), and the surface temperature of the substrate was monitored with a pyrometer.

### Processing for Liftoff

Gallium phosphide films on silicon were patterned by photolithography and the GaP was etched with a 5:1:1 mixture of deionized water, 97% sulfuric acid, and 30% hydrogen peroxide, similar to approaches previously used for GaAs etching^27^. Etch completion was judged by eye and confirmed by dipping in buffered hydrofluoric acid, owing to the hydrophobic nature of clean Si, whereas Si covered by GaP remained hydrophilic.

To form contacts, ohmic Ni/Zn/Au alloy^28^ contacts of 100 nm total thickness were evaporated through a shadow mask onto the p-type GaP film corners. After evaporation, the contacts were annealed and an additional 500 nm of silver was evaporated to protect the ohmic contact and define the thickness of the interlayer.

### Bonding Process

The sample and glass slide (Eagle XG 20/10, Coresix) were thoroughly cleaned with solvents and dried with N_2_, and baked at 180 °C for 5 minutes to dehydrate. SU-8 2002 was spun on both the sample and the glass slide at 3000 RPM for 30 seconds. If any particles are present distorting the resist, the surface was re-cleaned and dehydrated. Both the glass and sample were then soft baked for 2 minutes at 95 °C. The sample was brought into contact with the glass, then baked for 30 minutes in a convection oven at 95 °C. The sample was then placed in a Suss wafer bonder with 500 mbar of pressure at 95 °C. Finally, the SU-8 was hardened with a 1 minute flood exposure using 365 nm light through the glass and a 20 minute hot plate bake at 180 °C.

### Photoluminescence Measurements

Photoluminescence measurements were performed using a Coherent Libra Ti:sapphire laser, fed into an OPerA solo OPA, equipped with nonlinear optics capable of a variety of outputs. A 360 nm excitation pulse was chosen with an intensity of 20 nJ/pulse. A 364 nm long pass filter was used to block the primary beam and a streak camera was used as a detector to acquire spectral data.

### X-ray diffraction Measurements

X-ray diffraction measurements were performed using a Panalytical X’Pert Pro system equipped with a Cu X-ray anode paired to a hybrid monochromator (18 arcseconds resolution). All measurements were done after aligning to the Si substrate peak. 2θ-ω measurements were performed with a receiving slit before the detector, while rocking curves and reciprocal space mapping were done with a triple bounce Ge (220) analyzer (12 arcseconds resolution).
